# Targeting Ferroptosis in Colorectal Cancer

**DOI:** 10.3390/metabo12080745

**Published:** 2022-08-12

**Authors:** Xiaojie Liang, Zhihuan You, Xinhao Chen, Jun Li

**Affiliations:** 1 Department of General Surgery, The Affiliated Jiangning Hospital of Nanjing Medical University, Nanjing 211100, China; 2 Department of Cardiology, The Affiliated Jiangning Hospital of Nanjing Medical University, Nanjing 211100, China; 3 Department of Hepatobiliary and Pancreatic Surgery, The Affiliated Jiangning Hospital of Nanjing Medical University, Nanjing 211100, China

**Keywords:** ferroptosis 1, colorectal cancer 2, iron ions 3, lipid peroxides 4

## Abstract

Ferroptosis is a unique way of regulating cell death (RCD), which is quite different from other programmed cell deaths such as autophagy. It presents iron overload, accumulation of reactive oxygen species (ROS), and lipid peroxidation. A ferroptotic cell usually has an intact cell structure as well as shrinking mitochondria with decreased or vanishing cristae, concentrated membrane density, and ruptured outer membrane. Recently, increasing investigations have discovered that tumor cells have a much greater iron demand than the normal ones, making them more sensitive to ferroptosis. In other words, ferroptosis may inhibit the progress of the tumor, which can be used in the therapy of tumor patients, especially for those with chemotherapy resistance. Therefore, ferroptosis has become one hot spot in the field of tumor research in recent years. Colorectal cancer (CRC) is one common type of gastrointestinal malignancy. The incidence of CRC appears to have an upward trend year by year since the enhancement of living standards. Although surgery and chemoradiotherapy have largely improved the prognosis of patients with CRC, some patients still appear to have severe adverse reactions and drug resistance. Moreover, much research has verified that ferroptosis has a necessary association with the occurrence and progression of gastrointestinal tumors. In this review, we provide a comprehensive evaluation of the main mechanisms of iron metabolism, lipid metabolism, and amino acid metabolism involved in the occurrence of ferroptosis, as well as the research progress of ferroptosis in CRC.

## 1. Introduction

Ferroptosis is a newly defined programmed cell death, which was officially named by Stock Well in 2012 [1]. Different from necrosis, apoptosis, and autophagy, ferroptosis cannot be inhibited by regulation cell death (RCD) inhibitors but can be suppressed and even reversed by antioxidants and iron chelators featuring iron overload, accumulation of reactive oxygen species (ROS), and lipid peroxidation [2,3]. Excess intracellular iron can enhance lipid peroxidation, which in turn induces cell death, showing unique morphological and biological metabolic changes [4,5]. Existing studies have observed increased membrane density, ruptured outer cell membrane, and lack of chromatin condensation in ferroptotic cells with integral cell structure. Scientists also find that mitochondria shrink in volume, and cristae decrease or disappear in these cells [6]. Recent research has demonstrated that ferroptosis widely participates in various diseases, such as brain degeneration and heart failure [7,8]. Moreover, the activation of ferroptosis can suppress the proliferation of tumor cells, and it is expected that ferroptosis in tumor cells will become a potential direction of tumor therapy [9]. Hence, ferroptosis has attracted general interest and attention in the tumor research field. Nowadays, the proportion of gastrointestinal tumors, especially colorectal cancer (CRC), in new tumor patients has been increasing. CRC has become the fourth most commonly diagnosed tumor in both sexes and the second cause of tumor death [10]. Although current treatment for CRC still prefers radical surgical resection supplemented by radiotherapy [1] and chemotherapy, the balance between clinical efficacy and adverse effects remains a problem. There is still a lack of precise therapeutic targets. In our review, we summarize multiple mechanisms of research about ferroptosis in CRC, exploring the possibility of applying ferroptosis in tumor therapy.

## 2. Crucial Pathways and Regulators of Ferroptosis

**Figure 1:**
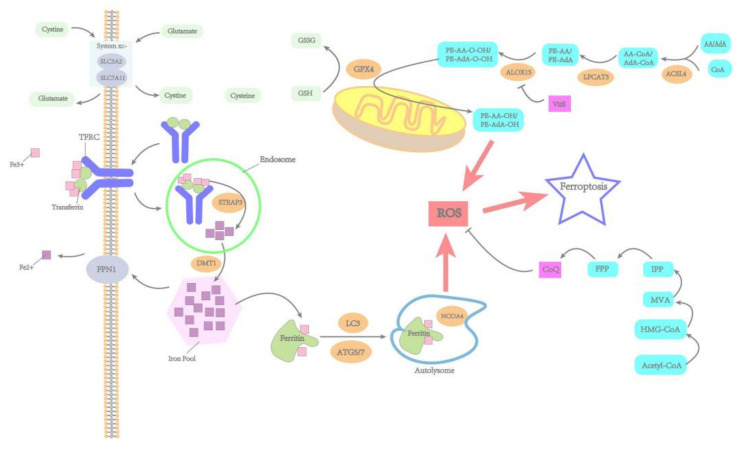
Crucial pathways of ferroptosis.

Ferroptosis is a unique kind of RCD involved in a variety of biological processes [4]. It is mainly regulated by the following mechanisms: iron metabolism pathways, lipid metabolism pathways, amino acid metabolism pathways, and so on. The occurrence of ferroptosis can be promoted by ROS generated by the Fenton reaction, polyunsaturated fatty acids (PUFAs), and lipid peroxidation, whereas system xc^-^/glutathione (GSH)/glutathione peroxidase 4 (GPX4) axis suppresses ferroptosis by affecting the redox state of cells [6]. Moreover, coenzyme Q (CoQ) in its reduced form can also inhibit ferroptosis by suppressing lipid peroxidation. In short, the key mechanism of ferroptosis lies in the accumulation of iron-dependent ROS, which leads to lipid peroxidation. The following Figure 1 is a detailed description of the processes.

Crucial pathways in ferroptosis are mainly involved in iron metabolism, amino acid metabolism, and lipid metabolism. Transferrin-transferrin receptor (TFRC) complex loaded with iron are internalized through endosomes and then release iron (Fe^2+^) into the cytoplasm via divalent metal transporter-1 (DMT1, also named SLC11A2). Ferritin combined with Fe^2+^ can degrade through ferritinophagy, which is mediated by nuclear receptor coactivator 4 (NCOA4) and release Fe^2+^. Fe^2+^ can be exported outside the cell mainly through ferroportin1 (FPN1, also named SLC40A1) in the membrane. The cystine/glutamate transporter (also called system xc^-^) can import cystine into the cell and export glutamate out of the cell. Cystine that enters the cell can be oxidized to cysteine and used to synthesize glutathione (GSH). The antioxidant system of GSH and glutathione peroxidase 4 (GPX4) can effectively protect cells from ferroptosis. Acyl-CoA synthetase long-chain family member 4 (ACSL4) and lysophospholipid acyltransferase 3 (LPCAT3) can promote the formation of phospholipids containing polyunsaturated fatty acids (PUFA-PLs).

### 2.1. Iron Metabolism

Iron is essential for numerous biological processes. Under normal circumstances, extracellular iron ions form a complex with transferrin (TF), which may be transported into the cell by binding to transferrin receptor 1 (TFR1) in the cell membrane [11]. It can be stored in the form of ferritin if there is too much iron in the cell, and iron ions will be released from ferritin and then transported out of the cell by FPN1 when the body needs iron. Thus, iron is one of the prerequisite factors for ferroptosis. On the one hand, the uptake, release, transport, and other activities of iron ions are all important conditions that affect the occurrence of ferroptosis. Intracellular irons mainly depend on the storage and export of iron. In contrast, iron metabolism can be regulated by certain forms of iron in the cell. For example, TF and TFRs regulate the transfer-in and transport of iron ions, and ferroportin, which is called solute carrier family 40 member 1 (SLC40A1) as well, mainly regulates the transfer-out of iron ions. Ferritin, including ferritin light chain 1 (FTL1) and ferritin heavy chain 1 (FTH1), can also regulate free iron by changing iron storage. On the other hand, the occurrence of ferroptosis is closely associated with ferritinophagy. By regulating iron metabolism, autophagy may increase the sensitivity to ferroptosis inducers. Ferritinophagy, a specific autophagic degradation of ferritin, can increase the cells’ sensitivity to iron ions by regulating intracellular iron ion transport, and it can be adjusted by a specific receptor named nuclear receptor coactivator 4 (NCOA4) [12,13,14,15,16]. NCOA4 combines with ferritin through the FTH1 subunit forming a complex after recognition, then transports iron ions to autophagosomes for lysosomal degradation before releasing them into the cytoplasm as free iron. However, it was found to cause only minor differences in ferroptosis if NCOA4 was knocked out in colon cancer cells, indicating that ferritinophagy may not be a critical factor in the process of ferroptosis in colon cancer cells [17]. Moreover, autophagy leads to ferroptosis by degrading ferritin in tumor cells mediated by multiple autophagy-related genes (ATG) [18]. Among them, ATG5 and ATG7 are indispensable for the formation of autophagosomes [19,20]. Studies have proved that active autophagy can degrade ferritin to increase the level of free iron, leading to oxidative damage caused by the Fenton reaction [21,22]. Another key iron regulatory protein named hepcidin, which is encoded by the gene HAMP is thought to affect inflammation and iron homeostasis in the body. It has been found that hepcidin inhibits ferroptosis by increasing FTH expression in LPS-induced ARDS [23]. In addition, hepcidin can promote ferroptosis through iron metabolism with DMT1 signaling to activate early brain injury after subarachnoid hemorrhage [24].

### 2.2. Amino Acid Metabolism

Recent research has confirmed that abnormal amino acid metabolism relates closely to ferroptosis [25]. System xc^-^ is a transmembrane protein complex including two subunits, solute carrier family 7 member 11 (SLC7A11) and solute carrier family 3 member 2 (SLC3A2), which can transport endogenous glutamate to the outside of the cell while transporting exogenous cystine into the cell at the same time. It would enhance the synthesis of intracellular glutathione (GSH) so as to decrease oxidative damage and inhibit the occurrence of ferroptosis. As for GSH, it is a small molecule active peptide constituted by glutamic acid, cysteine, and glycine. Its sulfhydryl structure can be oxidized and dehydrogenated to form oxidized glutathione, making GSH protect cells from oxidation as an important antioxidant in the body. One research has discovered that knockout of system xc^-^ in mice can protect them from neurotoxic damage caused by the accumulation of glutamate [26]. Therefore, the accumulation of extracellular glutamate is also considered to be one of the inducers of ferroptosis.

Glutathione peroxidase 4 (GPX4), which is the sole member of the GPX family, may reduce peroxide in lipid membranes, and its antioxidant effect is significantly higher than any others in the family [27]. GPX4 is known as a type of selenoprotein that can specifically and efficiently remove phospholipid hydrogen peroxide and other small molecular peroxides, thereby suppressing ferroptosis caused by massive ROS accumulation. Studies have improved that tumor cells with drug resistance are more sensitive to GPX4 [28]. Moreover, the main inhibitors of GPX4, including RAS-selective lethal 3 (RSL3), FIN56, DP12, etc., can result in cellular lipid peroxidation, ROS accumulation, and finally, ferroptosis through directly binding to GPX. Knockout of the GPX4 gene can obtain the same result [29]. In addition, GPX4 can be indirectly inhibited by the system xc^-^ inhibitor Erastin accompanied by ROS accumulation, which ends with ferroptosis as well. Hence, GPX4 is thought to be a vital regulator of ferroptosis.

### 2.3. Lipid Metabolism

Lipids form the structural basis of cell or organelle membranes [30]. In normal cells, lipid oxidation and reduction are in a state of dynamic equilibrium, but when cells become cancerous or have the intervention of exogenous factors, this balance will be broken. Ferroptosis is a new kind of cell death caused by the extra oxidation of phospholipids containing PUFAs, which are abundant in cell membranes [31,32]. PUFAs are lipid peroxidation’s main substrates, which may greatly destroy the structure and function of the cell membrane in ferroptosis, while monounsaturated fatty acids (MUFAs) suppress ferroptosis by inhibiting lipid peroxidation [33]. Thus, the abundance of PUFAs determines the extent of lipid peroxidation and the difficulty with which ferroptosis occurs. Acyl-CoA synthetase long-chain family member 4 (ACSL4) and lysophospholipid acyltransferase 3 (LPCAT3) are both the key enzymes that affect PUFA synthesis in phospholipid membranes, and their inhibition or deficiency leads to cellular resistance to ferroptosis [34,35]. ACSL4, LPCAT3, and PUFA can act together to esterify arachidonic acid or adrenic acid into phosphatidylethanolamines (PEs) and, finally, generate lipid hydroperoxide with lipoxygenase (LOXs). The decomposition products may destroy protein, make the plasma membrane shrink, and its curvature increase, further inducing ferroptosis [34].

### 2.4. CoQ10 Related Pathways

Ubiquinone (also known as coenzyme Q, CoQ) is a sort of endogenous lipid-soluble antioxidant in cells, including reduced and oxidized forms. The conventional metabolic pathways of CoQ10 consider that the reduced form can prevent the excessive oxidation of proteins, lipids, and DNA [36]. While recent studies have found that apoptosis-inducing factor mitochondria-associated 2 (AIFM2) inhibits ferroptosis regulated by reduced CoQ10. AIFM2 was originally described as an autophagy-promoted gene yet renamed ferroptosis suppressor protein 1 (FSP1) with the understanding of ferroptosis deepened. FSP1 generates ubiquinol, a reduced form of CoQ10, through NAD(P)H in order to further regulate lipid peroxidation [37]. The FSP1/CoQ/NAD(P)H pathway may serve as a parallel system in addition to the independent GPX4 system, which cooperates with GPX4 and GSH to suppress the occurrence of phospholipid peroxidation and ferroptosis [38]. Some studies have also found that AIFM2 can block Erastin/Sorafenib/RSL3-induced ferroptosis in tumor cells by an independent mechanism of ubiquinol [39]. Moreover, this ferroptosis resistance is associated with endosomal sorting complexes required for transport (ESCRT)-III recruitment based on a plasma membrane repair mechanism.

### 2.5. Other Pathways and Regulatory Factors

So far, the symbolic mechanism of ferroptosis remains unclear and needs more exploration. On the basis of current research, pathways such as MUC1-C/xCT [40,41,42], p53/SAT1/ALOX15 [43], p62/Keap1/Nrf2 [44,45,46,47], and ATG5/ATG7/NCOA4 [19] can adjust intracellular iron ions and ROS thus regulate ferroptosis to some extent. Moreover, current common inducers of ferroptosis include Erastin, RSL3, and inhibitors include ferrostastin-1 (Fer-1), deferoxamine (DFO), and liproxstatin-1 (Lip-1) [48,49,50,51,52]. Among the above pathways, the classic tumor suppressor p53 shows a crucial role in reactive oxygen species (ROS)-mediated ferroptosis. It can mediate ferroptosis by inhibiting the expression of SLC7A11, which may contribute to suppressing the tumor in turn [53]. Moreover, the knockdown of p53 completely abolished the inhibition of SLC7A11 [54].

## 3. Targeting Ferroptosis in Tumors

### 3.1. Targeting GSH-GPXs System

As one type of classic chemotherapeutic drug, Cisplatin was found to be a ferroptosis inducer causing the depletion of reduced GSH and the inactivation of GSH peroxidase, which played an important role in tumor ferroptosis. Moreover, it may show better antitumor results if Cisplatin was used in combination with Erastin [55]. Moreover, a new type of agent based on arginine-rich manganese silicate nanobubbles (AMSNs) was reported to induce ferroptosis by depleting GSH efficiently and inactivating GPX4, which can obtain a significant tumor suppression result then. This strategy targeting the depletion of GSH by certain nanomaterials will provide creative thoughts for the design of tumor-targeted therapy drugs [56]. In addition, Sulfasalazine inhibits system xc^-^ in a similar manner to Erastin and has been used in the clinical treatment of lymphoma, small-cell lung cancer, and prostate cancer [57]. Al-tretamine induces ferroptosis by inactivating GPX4 and is currently approved for clinical use in the treatment of ovarian cancer [58]. The tumor suppressor BRCA1-associated protein 1 (BAP1) is a deubiquitinase that removes histone 2A ubiquitination and was found to suppress SLC7A11 transcription probably through deubiquitination of histone 2A monoubiquitination (H2Aub) at lysine 119 on the SLC7A11 gene, leading to decreased cystine import, reduced GSH synthesis and then enhanced ferroptosis. Thus targeting BAP1 may enhance the effect of tumor suppression [59].

### 3.2. Targeting Lipid Peroxidation

Sorafenib is identified as an inhibitor of oncogenic kinases, also as a ferroptosis inducer approved by the FDA for the treatment of certain advanced cancers, including hepatocellular carcinoma, by targeting system xc^-^ [60]. Sorafenib was also found to induce ferroptosis in different cancer cell lines [61]. It was shown that metallothionein (MT)-1G may be a promising therapeutic target of Sorafenib resistance in human HCC cells. Knockdown of MT-1G increases glutathione depletion and lipid peroxidation, which contributes to Sorafenib-induced ferroptosis. Therefore, it would be a highly efficient strategy to block MT-1G expression so as to promote the anticancer effect of Sorafenib by inducing ferroptosis [62]. A recent investigation found that Apatinib may induce lipid peroxidation through GPX4 mediated by the transcription factors Sterol regulatory element-binding protein-1a (SREBP-1a), then lead to ferroptosis of GC cell, even the multi-drug-resistant GC cell [63]. In one study, a Black Hole Quencher (BHQ)-based fluorescence “off-on” nanophotosensitizer complex assembly (CSO-BHQ-IR780-Hex/MIONPs/Sor) was developed. CSO-connected BHQ-IR780-Hex and -loaded magnetic iron oxide nanoparticles (MIONPs) and Sorafenib (Sor) formed a concise functionalized delivery system, where the xCT/GSH/GPX-4 system was triggered, and lipid peroxidation burst when NIR irradiation was given to CSO-BHQ-IR780-Hex/MIONPs/Sor-treated cells with the iron supply increased [64]. It may provide ideas about solving low efficiency and side effects that ferroptosis therapy nanodevices have brought.

## 4. Role of Ferroptosis in CRC

CRC is a very common malignant tumor of the digestive system. Although a variety of chemotherapy drugs such as oxaliplatin and fluorouracil have been applied in the treatment of CRC, its mortality rate is still very high. In recent years, the research focused on ferroptosis in the field of oncology has been quite intensive, and there are also many scientific achievements that reveal the relationship between CRC and ferroptosis [65].

It is found that Apatinib can aim at the ELOVL6/ACSL4 signal pathway so as to induce ferroptosis in CRC [66]. Another study also discovered that LPCAT3 is downregulated and closely associates with the poor prognosis of CRC patients, suggesting that certain pathways of ferroptosis have molecular targets for CRC therapy. Scientists find that the copper chelator elesclimol has anticancer effects, and its induced copper chelation inhibits CRC through ferroptosis [67]. Lipocalin 2, a siderophore protein that regulates iron homeostasis, is upregulated in multiple tumor types. When overexpressed, it can reduce ferroptosis by reducing intracellular iron levels and stimulating the expression of GPX4 and xCT, leading to the resistance to 5-Fluorouracil (5-FU) in colon cancer cells both in vitro and in vivo [68]. Therefore, it can be thought of as a potential therapeutic target, and its corresponding monoclonal antibody may become the basis for the potential treatment of patients with colon cancer who do not respond to chemotherapy. In addition, miRNA-15a-3p has been proved to positively regulate ferroptosis by directly targeting GPX4 in CRC [69]. Colorectal cancer stem cells (CSCs) are always considered to be the origin of CRC progression. Moreover, it is confirmed that dichloroacetate (DCA) can reduce the stemness of CRC cells dependent on dose, which would be demonstrated by reducing the expression of stemness markers, the formation of tumor spheroid, and the ability of cell migration. Moreover, DCA can chelate iron in lysosomes and promote ferroptosis to reduce CRC cell stemness [70]. Erastin was proved to be an inhibitor of SLC7A11, which has significantly stronger cytotoxic effects on CSCs through in vitro and in vivo experiments and can alleviate chemoresistance in CSCs [71]. This study implicates that CSCs are more sensitive to ferroptosis, which can be used to suppress colorectal cancer progression and chemoresistance as a potential target. Another research implies that regulation of GPX4 by SRSF9 is a critical mechanism driving CRC tumorigenesis as well as resistance to Erastin-induced ferroptosis, which may provide a new idea to enhance the sensitivity to Erastin in CRC cells [72]. Specifically, SFRS9 becomes an inhibitor of ferroptosis by up-regulating the expression of GPX4 protein, while down-regulating SFRS9 may be an effective method for the treatment of CRC. TP53-induced glycolysis and apoptosis regulator (TIGAR) is a p53-inducible gene that may control metabolism and protect cells from apoptosis. TIGAR is identified as a potential regulator of ferroptosis resistance in CRC through the ROS/AMPK/SCD1 signal pathway, and its knockdown significantly increases the production of lipid peroxidation products and makes CRC cells more sensitive to Erastin-induced ferroptosis. The block of TIGAR also inhibits SCD1 expression dependent on redox reaction and AMPK. In addition, some researchers think that it may be an effective method of cancer therapy to induce ferroptosis by inhibiting system xc^-^, the specific cystine/glutamate antiporter. In numerous screening experiments, they found that a type of benzopyran derivative named 2-imino-6-methoxy-2H-chromene-3-carbothioamide (IMCA) significantly can suppress the cell viability of CRC cells with organ toxicity negligible [73]. Further research has revealed that IMCA obviously leads to ferroptosis in CRC by the accumulation of ROS resulting from lower expression of SLC7A11 and reduced content of cysteine and glutathione, while overexpression of SLC7A11 significantly weakens IMCA-induced ferroptosis. Furthermore, IMCA regulates the AMPK/mTOR/p70S6k signal pathway related to SLC7A11 and ferroptosis, which reveals that IMCA may be applied in the therapy of CRC. Furthermore, studies have confirmed GCH1/BH4 metabolism as an emerging ferroptosis resistance mechanism in CRC. To be more specific, blocking GCH1/BH4 promotes ferroptosis induced by Erastin via ferritinophagy, suggesting that GCH1 inhibitors combined with Erastin are probably a novel therapeutic strategy for CRC [74]. Unexpectedly, knockdown of GCH1 fails to enhance RSL3-induced ferroptosis in CRC. Moreover, the autophagy inhibitor can reverse the resistance to Erastin in GCH1-knockdown cells. In addition, cetuximab, a monoclonal antibody targeting the epidermal growth factor receptor (EGFR), is found to promote ferroptosis induced by RSL3 via suppressing Nrf2/HO-1 signal pathway in KRAS mutant CRC cells, which was lately confirmed in a nude mouse model [75,76]. The research has explained that cetuximab enhances the cytotoxic effect of RSL3 on KRAS mutant CRC cells as well as RSL3-induced ferroptosis by activating p38 MAPK and inhibiting the Nrf2/HO-1 pathway [77]. Moreover, it has been proved that cetuximab can weaken carbohydrate metabolism by suppressing glucose uptake and glycolysis instead of increasing ROS accumulation slowly but progressively, and vitamin C (VitC) can cause ferroptosis by blocking iron homeostasis [78]. VitC combined with cetuximab coordinates a type of lethal metabolic cell death triggered by ATP depletion and oxidative stress, which decreases acquired resistance to anti-EGFR antibodies. For high-dose VitC is safe in tumor patients, this may bring a thoughtful idea for patients with CRC who develop resistance to anti-EGFR therapy. A new study indicates that a type of natural product, β-elemene, is a novel ferroptosis inducer that can improve the sensitiveness of KRAS mutant CRC cells combined with cetuximab by promoting ferroptosis and suppressing EMT [79]. Another new ferroptosis inducer called talaroconvolutin A (TalaA) kills CRC cells dependent on dose and time. However, TalaA cannot induce apoptosis but strongly triggers ferroptosis, and its effect on inhibiting colorectal cancer cells via ferroptosis is much better than Erastin, which is a famous ferroptosis inducer, making it a new potential choice for CRC therapy [80]. The following Table 1 is a generalization of ferroptosis-related genes and their respective mechanisms.

## 5. Role of Ferroptosis in Other Intestinal Diseases

Extra iron in the intestine usually generates ROS through the Fenton and Haber–Weiss reactions, thereby triggering oxidative stress such as lipid peroxidation in intestinal epithelial cells (IECs), which can induce ferroptosis and ultimately lead to the occurrence of intestinal diseases. In addition to CRC, the progression of inflammatory bowel disease is also closely associated with ferroptosis [88].

Inflammatory bowel disease, including ulcerative colitis (UC) and Crohn’s disease (CD), is an immune-related and currently incurable chronic disease that clinically manifests progressive inflammatory damage to the intestinal mucosa [89]. Currently, most researches focus on the interaction among the commensal microbiota, IECs, and immune system. Some scientists have discovered that both patients with UC and a colitis-induced mouse model significantly inhibit endoplasmic reticulum stress signals to induce ferroptosis and exacerbate the inflammatory response. They also find that the phosphorylation of the nuclear factor kappa Bp65 subunit (NF-kBp65) inhibits ER stress-mediated IEC ferroptosis to alleviate UC, which can be regarded as a potential therapeutic target [90,91]. Moreover, some researchers established UC’s animal model induced by dextran sulfate sodium (DSS) and found that inhibiting ferroptosis can effectively improve DSS-induced UC, which is involved in blocking the Nrf2/HO-1 signal pathway [92]. In a recent study, curculigoside (CUR), a natural ingredient extracted from cactus, may prevent ferroptosis in UC by inducing GPX4, making it a potential drug for UC treatment [93].

As for CD, the latest study reports that OTSSP167, a specific inhibitor of maternal embryonic leucine zipper kinase (MELK), can significantly inhibit ferroptosis in intestinal tissue, macrophage infiltration, and M1 polarization in a DSS-induced mouse model, reducing the secretion of pro-inflammatory factors [94]. At the same time, the targeted suppression of MELK by OTSSP167 may also be a potential method to control the progression of CRC [95]. In addition, the ferroptosis inhibitor Fer-1 can alleviate the symptoms to some extent in the 2,4,6 trinitrobenzene sulfonic acid (TNBS)-induced mouse model [96]. The above findings provide a new promising therapeutic strategy for the treatment of CD, especially for those suffering from tolerance to existing immunosuppressants.

## 6. Conclusions

As a new kind of RCD, ferroptosis that depends on intracellular iron levels has become a tumor research hotspot in recent years. Current studies have demonstrated that the occurrence of ferroptosis is associated with abnormal iron metabolism, lipid metabolism, and amino acid metabolism [97]. Specific compounds that can regulate ferroptosis have been found in past studies, such as ferroptosis inducer Erastin, RSL3, and ferroptosis inhibitor Fer-1, Lip-1 [98]. However, the specific molecular mechanism of ferroptosis in tumorigenesis and progression is still not clear.

As one of the most common digestive system tumors in the population, CRC has attracted public attention due to its increasing incidence worldwide [99,100]. Several studies have indicated that ferroptosis plays an important role in CRC. This review aims to generalize the related research on ferroptosis in CRC.

So far, the specific mechanism of ferroptosis in the occurrence and progression of CRC remains unclear. Ferroptosis has two sides, just like a double-edged sword. On the one hand, tumor cells are more prone to ferroptosis than normal cells; thus, inducing ferroptosis of CRC cells can achieve a more targeted anticancer effect. On the other hand, inhibiting ferroptosis can it is quite effective for IECs to alleviate the non-infectious damage by ferroptosis suppression, and it is likely to further result in the occurrence of CRC under the long-term intestinal inflammatory immune microenvironment. Nowadays, using some specific compounds or drugs such as cetuximab can reduce the resistance to chemotherapy of patients with CRC via ferroptosis. It brings good news for many patients who obtain poor chemotherapy results after surgery or who are resistant to chemotherapy drugs. Although there are various methods to detect ferroptosis nowadays, such as the detection of ferric ions and ROS, lacking uniform standards and direct detection methods for ferroptosis is still a troublesome problem. In addition, ferroptosis-related genes have been proposed as biomarkers for predicting CRC, but new prognostic models based on these genes need further validation [101].

Nowadays, there is much research focusing on the regulations of ferroptosis as well. Some focus on a certain protein, such as hepcidin mentioned above, which may also be a possible therapeutic means of targeting ferroptosis. Some keep an eye on the drugs that have been used clinically, such as Sorafenib, Sulfasalazine, and Altretamine mentioned above. Some studies pay attention back to the classic tumor suppressor p53, which is famous for its ability to induce cell cycle arrest, apoptosis, and senescence to suppress tumors, and find that activation of p53 sensitizes cells to ROS and then triggers ferroptosis [102]. It may shed light on considering a new role of p53 in tumor ferroptosis.

Even more surprising is the huge potential role of macrophages in tumor ferroptosis. Tumor-associated macrophages (TAMs) act as double-edged swords in the tumor immune microenvironment (TIME) based on two different cellular polarizations and also have double-edged effects on ferroptosis. M1 TAMs can reduce the expression of SLC3A2 and SLC7A11 to induce ferroptosis then. M1 TAMs can also accelerate the Fenton reaction by providing superoxide, leading to excessive ROS production, which in turn promotes tumor ferroptosis. In contrast, M2 TAMs can indirectly inhibit ferroptosis by inactivating CD8+ CTLs or by suppressing PD-L1 [103]. Therefore, TAM is one of the highly valuable targets for increasing tumor sensitivity to ferroptosis-induced immunotherapy, which provides a possible solution for immunotherapy resistance.

In conclusion, ferroptosis is a promising research direction, providing several targets for many diseases, including CRC. Therefore, ferroptosis should obtain more emphasis and further studies as a new therapeutic target in the field of CRC.

## Figures and Tables

**Table 1:** Mechanisms of ferroptosis-related genes in CRC.

Ferroptosis-Related Genes	Mechanisms
ACSL4	Apatinib promotes ferroptosis in colorectal cancer cells by targeting ELOVL6/ACSL4 signaling [66].
LPCAT3	LPCAT3 inhibitors remodel the polyunsaturated phospholipid Content of human cells and protect from ferroptosis [81].
LCN2	Lipocalin 2 expression promotes tumor progression and therapy resistance by inhibiting ferroptosis in colorectal cancer [68].
GPX4	RSL3 drives ferroptosis through GPX4 inactivation and ROS production in colorectal cancer [82].
SLC7A11	Targeting SLC7A11 specifically suppresses the progression of colorectal cancer stem cells via inducing ferroptosis [71].
MiR-15a-3p	MiR-15a-3p regulates ferroptosis via targeting glutathione peroxidase GPX4 in colorectal cancer [69].
SFRS9	Inhibition of SRSF9 enhances the sensitivity of colorectal cancer to Erastin-induced ferroptosis by reducing glutathione peroxidase 4 expression [72].
TP53	Cullin-9/p53 mediates HNRNPC degradation to inhibit Erastin-induced ferroptosis and is blocked by MDM2 inhibition in colorectal cancer [83].
SCD1	TIGAR drives colorectal cancer ferroptosis resistance through ROS/AMPK/SCD1 pathway [84].
Nrf2/HO-1	Cetuximab promotes RSL3-induced ferroptosis by suppressing the Nrf2/HO-1 signaling pathway in KRAS mutant colorectal cancer [76].
GCH1	Blockade of GCH1/BH4 axis activates ferritinophagy to mitigate the resistance of colorectal cancer to Erastin-induced ferroptosis [74].
TMEM16F	Activating TMEM16F is a crucial component during ferroptotic cell death [85].
STEAP3	Human STEAP3 maintains tumor growth under hypoferric conditions [86].
OTUD1	OTUD1 plays a stimulatory role in iron transportation and highlights the importance of OTUD1-IREB2-TFRC signaling axis in host antitumor immunity [87].

Ferroptosis-related genes involved in CRC.
